# Anatomical study of the transfer of flexor digitorum superficialis nerve branch of median nerve to restore wrist extension and forearm pronation

**DOI:** 10.31744/einstein_journal/2019AO4489

**Published:** 2019-06-17

**Authors:** Edie Benedito Caetano, Luiz Angelo Vieira, Cristina Schmitt Cavalheiro, Marcel Henrique Arcuri, Rodrigo Guerra Sabongi

**Affiliations:** 1 Pontifícia Universidade Católica de São Paulo, Sorocaba, SP, Brasil.; 2 Pontifícia Universidade Católica de São Paulo, São Paulo, SP, Brasil.; 3 Escola Paulista de Medicina, Universidade Federal de São Paulo, São Paulo, SP, Brasil.

**Keywords:** Tendon transfer, Median nerve, Muscle, skeletal, Fingers, Wrist, Forearm, Pronation, Cadaver, Transferência tendinosa, Nervo mediano, Músculo esquelético, Dedos, Punho, Antebraço, Pronação, Cadáver

## Abstract

**Objective:**

To analyze the anatomical variations of the innervation of the flexor digitorum superficialis muscle and to determine if the branch of the median nerve that supply this muscle is connected to the branches to the extensor carpi radialis brevis and the pronator teres muscles, without tension, and how close to the target-muscles the transfer can be performed.

**Methods:**

Fifty limbs of 25 cadavers were dissected to collect data on the anatomical variations of the branches to the flexor digitorum superficialis muscle.

**Results:**

This muscle received innervation from the median nerve in the 50 limbs. In 22 it received one branch, and in 28 more than one. The proximal branch was identified in 22 limbs, and in 12 limbs it shared branches with other muscles. The distal branch was present in all, and originated from the median nerve as an isolated branch, or a common trunk with the anterior interosseous nerve in 3 limbs, and from a common trunk with the flexor carpi radialis muscle and anterior interosseous nerve in another. It originated distally to the anterior interosseous nerve at 38, in 5 on the same level, and in 3 proximal to the anterior interosseous nerve. In four limbs, innervation came from the anterior interosseous nerve, as well as from the median nerve. Accessory branches of the median nerve for the distal portion of the flexor digitorum superficialis muscle were present in eight limbs.

**Conclusion:**

In 28 limbs with two or more branches, one of them could be connected to the branches to the extensor carpi radialis brevis and pronator teres muscles without tension, even during the pronation and supination movements of the forearm and flexion-extension of the elbow.

## RESUMO

**Objetivo:**

Analisar as variações anatômicas da inervação do músculo flexor superficial dos dedos e determinar se o ramo do nervo mediano destinado a esse músculo pode ou não ser conectado aos ramos para os músculos extensor radial curto do carpo e pronador redondo sem tensão, e quão próximo dos músculos-alvo a transferência pode ser realizada.

**Métodos:**

Foram dissecados 50 membros de 25 cadáveres para coletar dados sobre as variações anatômicas dos ramos para o músculo flexor superficial dos dedos.

**Resultados:**

O referido músculo recebeu inervação do nervo mediano nos 50 membros. Em 22 recebeu um ramo, em 28 mais que um. O ramo proximal foi identificado em 22 membros e em 12 compartilhava ramos com outros músculos. O ramo distal estava presente em todos e desprendeu-se do nervo mediano como um ramo isolado ou de um tronco comum com o nervo interósseo anterior em 3 membros, e de um tronco comum com músculo flexor radial do carpo e nervo interósseo anterior em outro. Originou-se distalmente ao nervo interósseo anterior em 38, em 5 no mesmo nível e em 3 proximal ao nervo interósseo anterior. Em quatro recebeu inervação do nervo interósseo anterior, além daquela recebida pelo mediano. Ramos acessórios do nervo mediano para a porção distal do músculo flexor superficial dos dedos estavam presentes em oito membros.

**Conclusão:**

Nos 28 membros em que existam 2 ou mais ramos, 1 desses poderia ser conectado aos ramos para o músculo extensor radial curto do carpo e pronador redondo sem tensão, mesmo durante os movimentos de pronossupinação do antebraço e flexão-extensão do cotovelo.

## Introduction

The flexor digitorum superficialis (FDS) is the largest muscle in the forearm. It forms an intermediate muscular layer, between the superficial and deep groups. It receives innervation from the median nerve, but may also occasionally receive innervation from the anterior interosseous nerve (AIN).^(1,2)^ It has been described that, most commonly, FDS receives two or more branches from the median nerve (MN), and one of these branches can be used to reinnervate paralyzed muscles, particularly in brachial plexus lesions, since the distance and time required for the reinnervation of forearm muscles usually prevent recovery of function.^(2-5)^ Nerve damage treatment is based on primary nerve repair, nerve grafts, tendon transfers, and free transfer of muscles. However, there are nerve lesions that are not amenable to primary repair and for which grafts do not provide satisfactory results. These lesions include very proximal nerve injury; extensive area of injury, resulting in a long gap between nerve stumps; and idiopathic nerve palsy or neuritis, in which no healthy proximal nerve segment is available.^(2-4)^ In the case of brachial plexus lesions, with very large gaps between nerve endings, the axon regeneration time may be too long for them to reach the motor endplates of the target muscles before they become permanently resistant to reinnervation. This prolonged period of denervation leaves target muscles susceptible to irreversible degeneration and fibrosis of the motor endplates.^(2-4)^

Tendon transfers are performed as the first choice treatment for brachial plexus injuries, but they may be limited because the results are often inconsistent.^(5-7)^ Plate et al.,^(6)^ see advantages and disadvantages in nerve transfers, and consider them preferable over tendon transfers for the following reasons: nerve transfers may employ dispensable or redundant nerves, whereas tendon transfers require sacrifice of the donor muscle. Tendon transfers require more extensive muscle dissections, and calculating adequate length and tension during a tendon transfer procedure is difficult. Tendon transfers are often associated with joint stiffness, disorders of natural muscular biomechanics, fibrosis, and impaired vascularization. Availability of donor muscles is necessary to restore function of paralyzed nerves. The disadvantage of nerve transfers is the time required to reinnervate target muscles. Some authors recommend that a branch from the MN to FDS be transferred to the branch of the radial nerve that supplies the extensor carpi radialis brevis (ECRB) muscle, due to its synergistic relation with the wrist extensor muscles.^(4)^ Selection of donor nerves that have synergistic action with the receptor nerves facilitates subsequent cortical integration. The flexion of the wrist increases the passive tension of the finger extensor muscles, and thus causes the extension of the fingers, increasing the force of extension, whereas the extension of the wrist has the opposite effect and allows the fingers to flex passively.^(1,4,8)^ Redundant branches to the FDS muscle have also been used to restore forearm pronation, with excellent functional results.^(1)^ Previous anatomical studies showed that the innervation of the FDS muscle showed greater variability in relation to other forearm muscles innervated by MN, which also present great variability.^(9-13)^

## Objective

To determine the anatomical variations in innervation of the flexor digitorum superficialis muscle in 50 cadaveric limbs, and to evaluate whether the branches of the median nerve that supply the flexor digitorum superficialis muscle are connected to the branches leading to the receptor nerves, particularly in the extensor carpi radialis brevis and the pronator teres muscles, without tension, and how close to the target-muscles the transfer can be performed.

## Methods

The study was conducted at the Anatomy Laboratory of the *Faculdade de Ciências Médicas e da Saúde* of the *Pontifícia Universidade Católica*, Sorocaba Campus (SP). It was based on the dissection of 50 limbs of 25 adult cadavers, all males. All the specimens made available followed institutional ethical precepts, and the project was approved by the Research Ethics Committee under number 2.207.258, CAAE: 83985818.7.0000.5373. They were prepared by intra-arterial injection of a 10% formalin and glycerol solution. Each forearm was dissected with the elbow extended, the wrist in neutral position, and the forearm pronated. None of the cadavers presented evidence of deformities, previous surgical procedures or traumatic lesions in the studied area. All measurements were performed during the procedures directly on the anatomical pieces, with systematized material and methodology. We removed the skin and fascia from the distal third of the arm, forearm and wrist. Median nerve was identified in the arm and dissected from proximal to distal. The bicipital aponeurosis was sectioned. The pronator teres muscle was disinserted distally and retracted away. Tendons of the flexor carpi radialis (FCR) and palmaris longus (PL) muscles were sectioned in the distal third to facilitate the identification of their nerve branches. The MN branches for the pronator teres (PTM), FCR, PL, FDS muscles and the AIN with its branches for the flexor digitorum profundus (FDP), flexor policis longus (FPL) and pronator quadratus (PQ) muscles were dissected after longitudinal division of the FDS muscle and its fibrous arch, along the AIN in the forearm, from proximal to distal. The radial nerve was identified in the arm between brachialis (BM) and brachioradialis (BR) muscles. We identified the nerve branches for BM, BR, extensor carpi radialis longus (ECRL), ECRB muscles, the radial nerve superficial branch (RSNR), the posterior interosseous nerve (PIN) and its branches to the supinator muscle. With a digital caliper and a millimeter ruler, we measured the diameter and length of the branch for ECRB, PTM and FDS. Vascular structures were not preserved to facilitate dissection of the nerves. The following measurements were made: (1) Forearm length, from the center of a line between the medial and lateral epicondyles (intercondylar line) to the center of a line between the radial and ulnar styloid processes; (2) the distance from the medial epicondyle to the origin of the branch for FDS and PTM in MN; (3) the length of each muscle branch from its MN origin to the neuromuscular junction. We used, at certain stages of the dissection, a magnifying glass with 2.5x magnification. We use a millimeter ruler and a digital caliper to measure the length and diameter of donor and recipient nerves. Branches for FDS and PTM were measured in 14 limbs, and the branch for ECRB was evaluated in 30 limbs.

## Results

**Figure 1:**
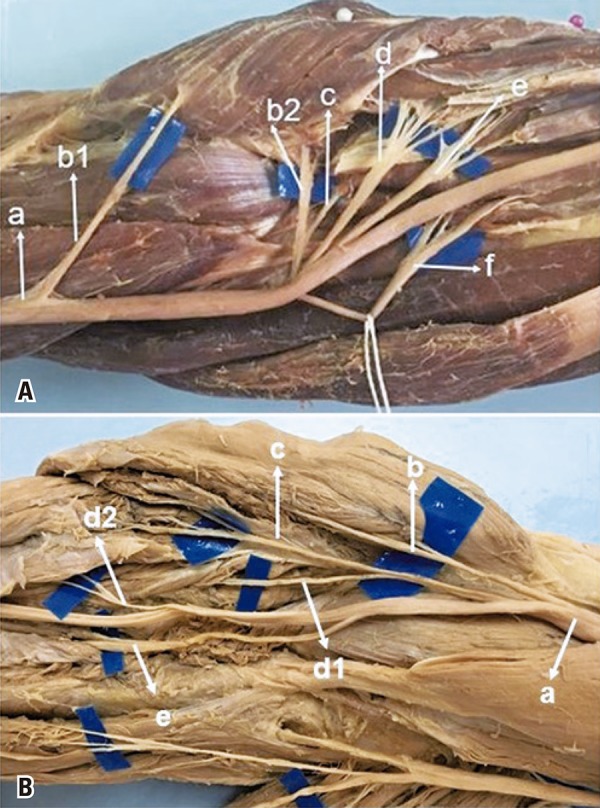
Innervation from the median nerve received by the flexor digitorum superficialis. (A) Median nerve (a), first branch for the pronator teres muscle (b1), second branch for the pronator teres muscle (b2), branch for the palmaris longus muscle (c), branch for the flexor carpi radialis muscle (d), branch for the flexor digitorum superficialis muscle (e), anterior interosseous nerve (f). (B) Median nerve (a), branch for the pronator teres muscle (b), branch for the palmaris longus muscle (c), branch for the flexor carpi radialis muscle (c), first branch for the flexor digitorum superficialis (d1), second branch for the flexor digitorum superficialis muscle (d2), anterior interosseous nerve (e), palmaris longus muscle absent

**Figure 2:**
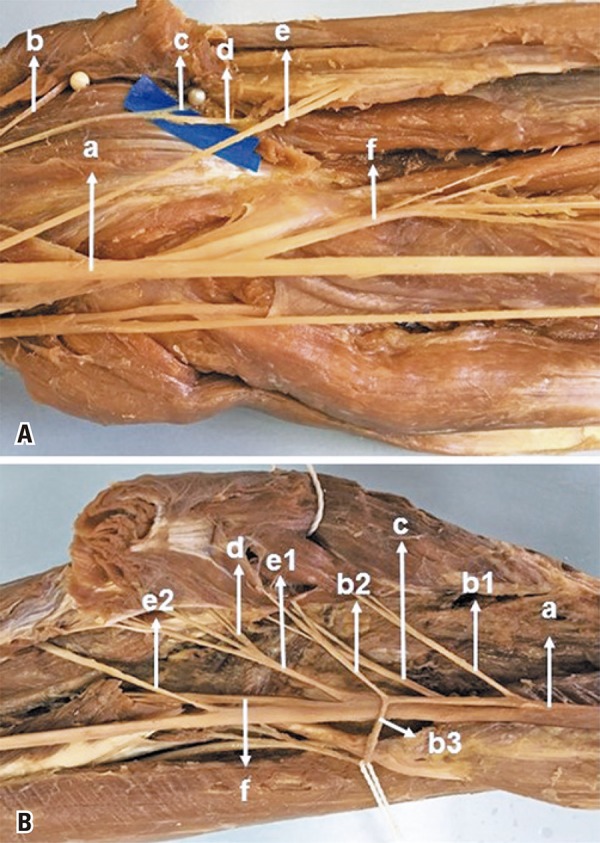
Anatomical relation of the forearm muscles innervated by the median nerve. (A) Median nerve (a), branch for the pronator teres muscle (b), branch for the palmaris longus muscle (c), branch for flexor digitorum superficialis (d), branch for flexor carpi radialis muscle (e), anterior interosseous nerve (f). (B) Median nerve (a), first branch for the pronator teres muscle. (b1), second branch for the pronator teres muscle (b2), third branch for the pronator teres muscle (b3), branch for the palmaris longus muscle (c), branch for the flexor carpi radialis muscle (d), branch for the flexor digitorum superficialis muscle (e1), second branch for the flexor digitorum superficialis (e2), anterior interosseous nerve (f)

**Figure 3:**
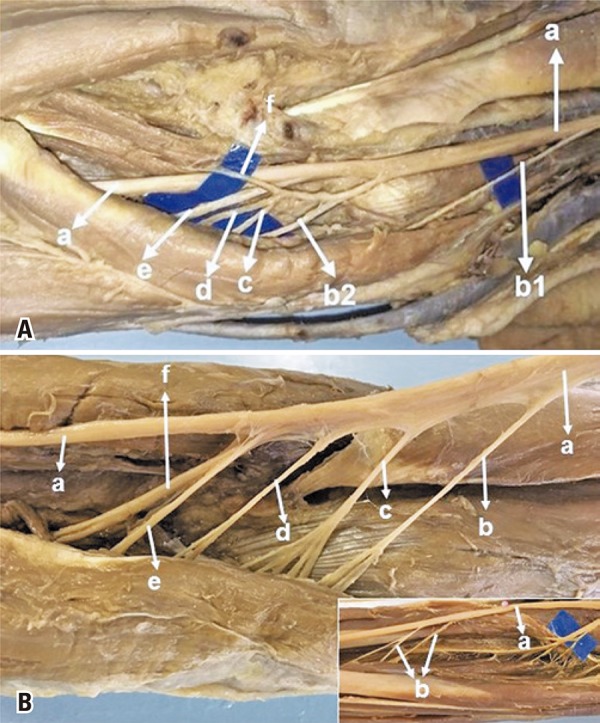
Anatomical variation in the innervation of the flexor digitorum superficialis. (A) Median nerve (a), first branch for the pronator teres muscle (b1), second branch for the pronator teres muscle (b2), branch for the palmaris longus muscle (c), branch for the flexor carpi radialis muscle (d), anterior interosseous nerve (e), branch for the flexor digitorum superficialis (f). (B) Median nerve (a), branch for the pronator teres muscle (b), branch for the palmaris longus muscle (c), branch for the flexor carpi radialis muscle (d), branch for the flexor digitorum superficialis muscle (e), anterior interosseous nerve (f). In detail: median nerve (a), accessory branches for the flexor digitorum superficialis (b)

The FDS muscle received MN innervation in the 50 dissected limbs. In 22 (44%) it received only one branch (Figure 1A), in 28 (56%) it received more than one branch from MN (2 branches in 23, and 3 branches in 5) (Figure 1B). The proximal branch was identified in 22 limbs; in that, in 3 from a common trunk with branches to PL (Figure 2A), in 3 from a common trunk with FCR (Figure 2B), in 2 from a common trunk for PL and FCR, in 4 from a common trunk for PTM, PL, FCR (Figure 3A). The distal branch present in all limbs (100%) originated from the MN as an isolated branch or from a common trunk with the AIN in 3 limbs (6%) (Figure 3B), from a common trunk with FCR and AIN in 1 (2%). The distal branch originated from the median nerve distally to the AIN in 38 limbs (76%) (Figure 1B), in 9 (18%) at the same level as the AIC (Figure 1A), and in 3 (6%) proximal to the AIN. The proximal branch originated 3.8±1.0cm below the intercondylar line. The distal branch was located 6.5±2.7cm below this line. The mean length of the branches for the FDS was 3.2±1.8cm. In 4 limbs, the FDS muscle received innervation from the AIN besides the innervation received from the median. Median nerve accessory branches for the distal portion of the FDS were observed in 8 limbs (16%); in 5 (10%) of these it was the third branch (Figure 3B). The anatomical measurement results for the nerves are shown in table 1. We considered one of the branches for FDS as a possible donor for the ECRB and PTM receptor nerves. In this study, the mean diameter of the FDS was 1.6±0.5, the ECRB 1.4±07, the ECRL 1.5±0.6, and the PTM 1.5±0.6.

**Table 1:** Distribution of the branch for the flexor digitorum superficialis muscle and association with branches of other forearm muscles

Muscle	Branches			No associations	Branches					Multiple associations	Limbs	Absent
	
	1	2	3		PTM	PL	FCR	FDS	AIN			
FDS	22	23	5	34	----	3	3	----	3	PTM + PL + FCR (4)	50	0
										FCR + AIN (1)		
										PL + FCR (2)		

PTM: pronator teres muscle; PL: palmaris longus; FCR: flexor carpi radialis; FDS: flexor digitorum superficialis; AIN: anterior interosseous nerve

## Discussion

Previous anatomical studies show that the innervation of the FDS muscle has great variability, similarly to other forearm muscles innervated by MN. Flexor digitorum superficialis innervation is rather controversial in the literature (Table 2).^(1,10-17)^ Most of the authors consider the FDS muscle to be innervated by more than one branch from MN, and one of these branches may be used to reinnervate paralyzed muscles.^(1,3,4)^

**Table 2:** Median nerve branches for the flexor digitorum superficialis muscle, as per number of limbs, position regarding the intercondylar line, number of branches for the flexor digitorum superficialis muscle and association with branches of other muscles

Authors	Limbs (n)	Type of study	Humeral intercondylar line			Branches for FDS		Associated innervation n (%)
	
			Proximal	At level	Distal n (%)	1 branches n (%)	2 to 3 branches n (%)	
Tung et al.^(1)^	31	Anatomic	0	0	31 (100)	2 (26)	29 (94)	(88)
Gunther et al.^(10)^	20	Anatomic	-	-	20 (100)	15 (75)	3 (13.5)	2 (10)
Canovas et al.^(11)^	10	Anatomic	0	0	10 (100)	8 (80)	2 (20)	6 (60)
Chantelot et al.^(12)^	50	Anatomic	-	-	-	34 (68)	-	16 (32)
Ukrit et al.^(13)^	10	Anatomic	0	0	10 (100)	0	10 (100)	4 (60)
Ye et al.^(14)^	46	Anatomic	-		46 (100)	19 (41.3)	27 (58.7)	-
Unver Dogan et al.^(15)^	50	Anatomic	-	-	-	44 (88)	6 (12)	7 (14)
El Zawary et al.^(16)^	20	Anatomic	-	-	-	-	2 to 6	-
Parot et al.^(17)^	20	Anatomic	-	-	-	0	20 (100)	15 (75)
Present study	50	Anatomic	0	0	50 (100)	22 (44)	28 (56)	16 (32)

FDS: flexor digitorum superficialis muscle.

Lowe et al.,^(3)^ proposed the transfer of redundant medium-sized branches that supply the FDS muscle to reinnervate PIN, since the FDS branch is an antagonist to the finger extensors. The result of this procedure was unsatisfactory. Subsequently, Ray et al.,^(4)^ considering synergistic relations, transferred the FDS branch to the ECRB branch and obtained positive clinical results in 18 of the 19 patients. Ukrit et al.,^(13)^ transferred a redundant branch for FDS to ECRB in two patients with brachial plexus palsy of C5, C6, and C7 roots, obtaining excellent results. Davidge et al.,^(18)^ reported that most of their patients had excellent recovery, including finger and thumb extension movement, which had never been observed with tendon transfers or nerve grafts before. Garcia-López et al.,^(8)^ prefer the transfer to the ECRL muscle instead of the ECRB. They consider that dissection and identification of the branches for the ECRL are less laborious than for the ECRB. Since the ECRL tendon inserts more radially than the ECRB, they observed some radial deviation in wrist extension during early recovery. This was resolved soon after, as the extensor carpi ulnaris (ECU) became reinnervated by the PIN. In our measurement, the ECRB branch was the longest branch of the radial nerve in the elbow region. We identified a variable length (4.5±2.5cm), which could be sectioned at an appropriate location and directed to the donor nerve, reducing the distance between donor and recipient. Tung et al.,^(1)^ reported two cases of patients with traumatic loss of PTM function and successful reconstruction by transferring a redundant branch for FDS to the PTM branch. The transfer of the MN branch for FDS provides several advantages. First, it reduces the distance between the donor and recipient branches, facilitating a faster recovery than nerve suture or graft. Second, the branches of the FDS and ECRB are motor. Third, redundant branches are employed, maintaining the innervation and function of the FDS muscle.^(2,4)^ On the other hand, Kaufmann et al.,^(19)^ reported that the use of the FDS nerve may result in loss of strength in the fingers. Bertelli et al.,^(20)^ performed three procedures in young patients with lesions of the C5-C8 roots of the brachial plexus, all operated before completing 6 months of the trauma, transferring a branch from the FDS to the ECRB branch. Twenty four months after surgery, all patients regained some degree of active wrist extension. They concluded that the use of a branch for the FDS to obtain wrist extension after C5-C8 brachial plexus palsy led to limited recovery in terms of strength, range of motion and motor control. The most problematic limitation was the inability to maintain the wrist in a neutral position without flexing the fingers.

**Figure 4:**
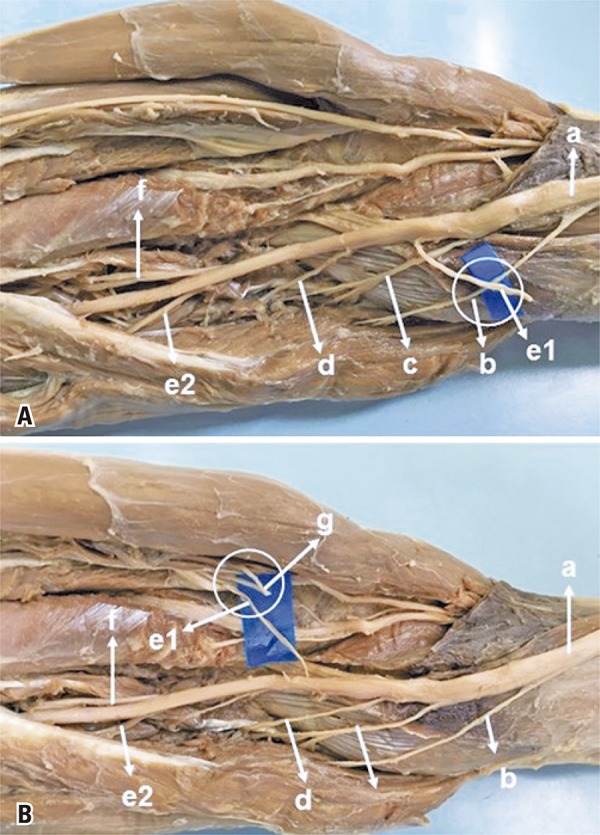
Transfer of the branch for the flexor digitorum superficialis to the pronator teres. (A) Median nerve (a), branch for the pronator teres muscle (b), branch for the palmaris longus muscle (c), branch for the flexor carpi radialis muscle (d), first branch for the flexor digitorum superficialis muscle (e1) transferred to the pronator teres muscle (b), (d), second branch for the flexor digitorum superficialis (e2), and anterior interosseous nerve (f). (B) Median nerve (a), branch for the pronator teres muscle (b), branch for the palmaris longus muscle (c), branch for the flexor carpi radialis muscle (d), first branch for the flexor digitorum superficialis (e1) transferred to the extensor carpi radialis brevis (g), second branch for the flexor digitorum superficialis muscle (e2), anterior interosseous nerve (f)

The innervation of the FDS by more than one branch was observed in 56% of the limbs that we dissected. The proximal branch appears to be the best choice for restoring wrist extension or forearm pronation because of the proximity to the receptor nerves.^(1)^ In 12 limbs of our study, it originated from a common trunk with branches to other muscles, from which it could be separated by internal neurolysis, or associated with the PL or FCR in the transfer, increasing the number of donor axons, which are also dispensable, since flexion of the wrist can be performed by the flexor carpi ulnaris. We reproduced the *in vivo* procedure *in vitro*, observing that in the 28 limbs in which there were more than one branch for the FDS, it was possible to connect one of the branches for the FDS with the branches for the PTM (Figure 4A) without tension, even during pronation and supination movements. The branches were sectioned at the neuromuscular junction and connected as close as possible to the muscular body of the receptor nerve. The “donor distal, recipient proximal” mantra is useful to remember.^(21)^ By sectioning the donor nerve as distally as possible and the receptor nerve as close as possible, the length of each nerve branch will be maximized to allow that the distal end of the donor nerve reaches the proximal end of the receptor nerve.^(21)^ Sukegawa et al.,^(2)^ recommend that the section of the branches depends on each situation and must be decided during the surgical procedure, identifying the connection point between the receiver and the donor, and the latter should be sectioned 5mm beyond this point, thus avoiding tension in the suture line. The mean diameter of the branches for FDS (1.6±0.5) is compatible with the mean nerve diameter for PTM (1.5±0.6). There was also no difficulty in connecting to the ECRB, which can easily be directed and connected to the FDS without tension (Figure 4B). The diameter of the branch for the ECRB (1.4±0.7) is not a problem, since several studies in the literature show that nerve transfers of branches with considerable diameter and nerve fiber differences provide good results. Some researchers believe that reinnervation of 20 to 30% of muscle fibers is compatible with normal muscle function.^(22,23)^ Jiang et al.,^(24)^ report that donor nerve axons can multiply and increase three to four times in number.

In this study, the distal branch originated from the median nerve distally to the AIN in 38 limbs (76%), in 9 (18%) at the same level as the AIN, and in 3 (6%) proximal to the AIN. Ye et al.,^(14)^ classified the relation between FDS and AIN into three types as follows: type I, in 21 of 46 limbs (45.7%), the distal branch of FDS and the AIN had the same origin from MN; type II, in 18 limbs (39.1%), the distal branch of FDS and the AIN had different origins in MN and the distal branch for FDS originated distally to AIN; and type III, in 7 limbs (15.2%), the distal branch of the FDS originated from the AIN. The determination of such branching patterns and their incidence have considerable clinical significance. For types I and III, the identification of these two branches is important during surgery, which should be handled with care. The proximity of the branches makes the dissection difficult, and some of them may be damaged.^(14)^

## Conclusion

The classic pattern of innervation of the flexor digitorum superficialis muscle (only one branch for the flexor digitorum superficialis) was identified in 22 limbs. In the 28 limbs in which there were two or more branches for the flexor digitorum superficialis muscle, one of these could be connected to the branches for the extensor carpi radialis brevis and the pronator teres muscles, with no tension, even during the pronation-supination and flexion-extension movements of the elbow.
